# Migraine in Older Adults: Age‐Related Clinical Features From the Italian RICe Registry

**DOI:** 10.1111/ene.70761

**Published:** 2026-10-14

**Authors:** Giulia Paparella, Luigi Francesco Iannone, Giulia Paparella, Grazia Sances, Roberto De Icco, Gloria Vaghi, Cristina Tassorelli, Andrea Burgalassi, Francesco De Cesaris, Alberto Chiarugi, Francesca Gabriele, Raffaele Ornello, Simona Sacco, Maria Pia Prudenzano, Martino Gentile, Damiano Paolicelli, Giorgia Dalla Volta, Marco Bolchini, Innocenzo Rainero, Elisa Rubino, Maria Albanese, Fabrizio Vernieri, Gianluca Coppola, Gianluca Avino, Edoardo Mampreso, Delfina Ferrandi, Marta Altieri, Stefania Battistini, Antonio Granato, Simona Guerzoni, Antonio Russo, Marina de Tommaso, the RICe Study Group, Paola Merlo, Alessandro Viganò, Massimo Alessandri, Lucia Princiotta Cariddi, Milena Taglioli, Mariarosaria Valente, Rosaria Rapisarda, Alessandro Tiezzi, Giovanna Ballerini, Giovanna Viticchi, Catello Vollono, Luisa Fofi, Antonio Montisano, Licia Grazzi, Eugenia Rota

**Affiliations:** ^1^ Neurophysiopathology Unit, Department of Translational Biomedicine and Neuroscience University of Bari Aldo Moro Bari Italy; ^2^ IRCCS Neuromed Pozzilli Italy; ^3^ Department of Biomedical, Metabolic, and Neural Science University of Modena and Reggio Emilia Modena Italy; ^4^ Headache Science and Neurorehabilitation Unit IRCCS Mondino Foundation Pavia Italy; ^5^ Department of Brain and Behavioral Sciences University of Pavia Pavia Italy; ^6^ Headache Center and Clinical Pharmacology Unit Careggi University Hospital Florence Italy; ^7^ Section of Clinical Pharmacology and Oncology, Department of Health Sciences University of Florence Florence Italy; ^8^ Department of Biotechnological and Applied Clinical Sciences University of L'aquila L'Aquila Italy; ^9^ Neurology Unit “L. Amaducci”‐Department of Translational Biomedicine and Neuroscience University of Bari Aldo Moro Bari Italy; ^10^ U.O. Neurologia, Istituto Clinico Città di Brescia Gruppo San Donato Brescia Italy; ^11^ Department of Neuroscience, Headache Center University of Torino Turin Italy; ^12^ Unit of Neurology, Department of Systems Medicine University of Rome Tor Vergata Rome Italy; ^13^ Headache and Neurosonology Unit Fondazione Policlinico Campus Bio‐Medico Rome Italy; ^14^ Department of Medicine and Surgery Università Campus Bio‐Medico di Roma Rome Italy; ^15^ Department of Medico‐Surgical Sciences and Biotechnologies Sapienza University of Rome Latina Italy; ^16^ SOC Neurologia, Ospedale Santo Stefano USL Toscana Centro Prato Italy; ^17^ Neurology‐Euganea, Health Unit Headache Centre Padua Italy; ^18^ S.C. Neurologia, Azienda Ospedaliera Universitaria Santi Antonio e Biagio Alessandria Italy; ^19^ Department of Human Neurosciences Sapienza University of Rome Rome Italy; ^20^ Department of Medical, Surgical and Neurological Sciences University of Siena Siena Italy; ^21^ Headache Center, Neurology Clinic, University Hospital of Trieste University of Trieste Trieste Italy; ^22^ Digital and Predictive Medicine, Pharmacology and Clinical Metabolic Toxicology, Headache Center and Drug Abuse, Laboratory of Clinical Pharmacology and Pharmacogenomics AOU Policlinico di Modena Modena Italy; ^23^ Headache Centre, Department of Advanced Medical and Surgical Sciences University of Campania “Luigi Vanvitelli” Naples Italy

**Keywords:** aging, elderly, headache, migraine, registry

## Abstract

**Background:**

Primary headaches, particularly migraine, are prevalent disorders insufficiently characterized in older adults. This multicentre study evaluated headache diagnoses, focusing on migraine, across age groups in headache centers participating in the Italian Registry for Headache Disorders (RICe).

**Methods:**

Using a post hoc design, we evaluated 3477 patients enrolled at 30 headache centers (2019–2025). Patients were stratified into young adults (18–30 years), adults (31–45 years), middle‐aged (46–59 years), and elderly individuals (≥ 60 years). Demographic and clinical migraine characteristics were collected. Group differences were assessed using *χ* ^2^ tests and Kruskal–Wallis ANOVAs.

**Results:**

Of 3477 patients, 644 (18.5%) were elderly, 1285 (36.9%) middle‐aged, 990 (28.5%) adults, and 558 (16.1%) young adults. Among migraine patients (*n* = 3013, 86.7%), diagnoses differed across age groups (*χ* ^2^(6) = 71.5, *p* < 0.001). Elderly individuals more frequently had chronic migraine (CM) (*z* = 4.18), whereas migraine with aura was more common in young and adult patients (*z* = 3.62 and 4.97, respectively). Medication‐overuse headache was more frequent in the elderly (*z* = 3.56), who reported more monthly migraine days but lower pain intensity and fewer severe attacks (all *p* < 0.02). Stress and physical activity were common triggers. Among patients with available treatment data, some differences in prior preventive treatment patterns were observed, with more frequent use of beta‐blockers and onabotulinumtoxinA in the elderly, whereas anti‐calcitonin gene‐related peptide (CGRP) monoclonal antibody prescriptions did not differ across groups.

**Conclusions:**

Older adults attending headache centers more frequently presented CM, with higher attack frequency, lower pain severity, greater medication overuse, and distinct triggers, supporting tailored diagnostic and therapeutic strategies.

## Graphical abstract

This multicentre study of 3477 patients from 30 Italian headache centres identified distinct age‐related patterns in migraine. Older adults more frequently presented with chronic migraine, higher monthly migraine frequency, lower pain intensity, and medication‐overuse headache, as well as distinct trigger and preventive treatment patterns. These findings highlight the need for age‐tailored diagnostic and therapeutic strategies for migraine in the growing older population (This figure was created by the authors using software of public domain, including Microsoft Excel 2021 [Office LTSC Professional Plus], Paint.NET v5.1.11, and OpenAI ChatGPT [Model GPT‐4o]; all components are original and do not contain previously published or copyrighted material).

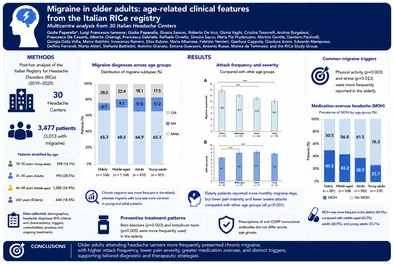

**Abbreviations**
ANOVA: analysis of variance
BoNT‐A: onabotulinumtoxinA
CCBs: calcium‐channel blockers
CGRP: calcitonin gene‐related peptide
CM: chronic migraine
CTTH: chronic tension‐type headache
ETTH: episodic tension‐type headache
FH: family history
ICHD‐3: International Classification of Headache Disorders, 3rd edition
IHS: International headache society
MA: migraine with aura
mAbs: monoclonal antibodies
MMD: monthly migraine days
MOH: medication‐overuse headache
MWA: migraine without aura
NRS: Numeric Rating Scale
NSAIDs: nonsteroidal anti‐inflammatory drugs
RICe: Italian Registry for Headache Disorders (Registro Italiano delle Cefalee)
SD: standard deviation
SEM: standard error of the mean
SNRIs: serotonin and norepinephrine reuptake inhibitors
SSRIs: selective serotonin reuptake inhibitors
TACs: trigeminal autonomic cephalalgias
TTH: tension‐type headache
WHO: World Health Organization

## 1 Introduction

Migraine is a highly prevalent and disabling neurological disorder across the lifespan, yet its presentation in older adults is still insufficiently characterized [1, 2, 3]. Although epidemiological studies suggest that overall migraine frequency declines with age, it may persist into later life, often with atypical or attenuated features [2, 4].

Indeed, migraine undergoes both qualitative and quantitative age‐related changes, including lower attack intensity, less prominent accompanying symptoms, and changes in pain localization [4, 5, 6]. At the same time, attack frequency may increase in a subset of patients, with a higher likelihood of chronification and medication overuse [2, 4, 5, 6, 7, 8, 9, 10, 11, 12]. Moreover, attenuated or atypical presentations may result in some older individuals fulfilling only the diagnostic criteria for probable migraine according to the International Classification of Headache Disorders, 3rd edition (ICHD‐3) [4, 13], further complicating diagnostic recognition. These age‐associated changes, together with the increasing burden of comorbidities and polypharmacy, may complicate diagnostic and therapeutic decision‐making in later life [2, 14], prompting some authors to propose that migraine in older adults be regarded as a separate entity [15].

Available evidence on older adults' migraine, however, is currently only reported from population‐based surveys or single‐center cohorts [4, 6, 13, 16, 17]. Although informative, such approaches may not fully capture the clinical variability encountered in real‐world headache care. Dedicated, multicenter registries provide an important methodological advantage, enabling the analysis of large, heterogeneous patient samples assessed according to standardized clinical protocols [18]. The Italian Registry for Headache Disorders (RICe) represents one of the most comprehensive national platforms for real‐world data collection across multiple headache centers [19, 20, 21]. Its structured and standardized framework allows systematic recording of demographic variables, diagnosis, headache characteristics, and treatment patterns.

A comprehensive understanding of the clinical heterogeneity of migraine in late life is increasingly relevant in the context of global population aging. Delineating age‐specific phenotypic patterns that may influence diagnostic accuracy and therapeutic decision‐making is therefore increasingly important. Aligning with these needs, the present study aims to characterize migraine across different age groups, with particular attention to older adults using a post hoc analysis of the RICe data. Unlike our previous single‐center study [4], the present investigation provides an external multicenter validation of those findings in a large, nationally representative real‐world cohort. Specifically, we aimed to: (i) describe the distribution of headache diagnoses in patients aged ≥ 60 years and compare this distribution with that observed in younger age groups; (ii) examine age‐related differences in migraine features.

## 2 Methods

### 2.1 Study Design

The RICe registry was established in 2019 to investigate the epidemiology of primary headache disorders in Italy and collects clinical data from patients evaluated at headache centers across different levels of care. For this post hoc analysis, we included prospectively collected data from 30 participating headache centers and analyzed demographic and clinical characteristics of outpatients attending either a first consultation or a follow‐up visit for headache between January 2019 and September 2025. For each patient, the index visit corresponded to the first clinical evaluation available in the registry. Patients were evaluated by expert headache specialists using a semi‐structured questionnaire to collect demographic and migraine‐related clinical information (see below). Headache diagnoses were established according to the ICHD‐3. All participants provided written informed consent for inclusion in the RICe registry, which was approved by the local Ethics Committee (CEAVC; Studio RICe protocol no. 14591_oss and subsequent amendments). The study was conducted in accordance with the Strengthening the Reporting of Observational Studies in Epidemiology (STROBE) guidelines.

For the group comparisons, we divided the patients into four age categories:

- Elderly (≥ 60 years);
- Middle‐aged (46–59 years);
- Adults (31–45 years);
- Young adults (18–30 years)

The WHO age limit was used to define the elderly group [2], and the middle‐aged group was intended to include most postmenopausal patients [4, 22, 23].

### 2.2 Patient Features and Variables Collected

The present study included all adult patients (age ≥ 18 years) enrolled in the RICe registry with a diagnosis of a primary or secondary headache disorder or painful cranial neuropathy according to the ICHD‐3 classification. Individuals aged < 18 years and those with incomplete demographic data were excluded.

For each patient, the recruiting center recorded:

– Unique patient identifier, sex, age, date of informed consent, and date of the clinical encounter;
– Headache concomitant diagnoses according to ICHD‐3 classification (up to five). For the purposes of the present study, only the primary headache diagnosis was considered. Medication‐overuse headache (MOH), which was systematically assessed for all major drug classes, was recorded as an additional diagnosis and analyzed separately.
– Migraine features including: age at migraine onset, presence of aura symptoms, migraine frequency (number of monthly headache days during the previous 3 months), pain intensity during the previous 3 months (assessed both by the Numeric Rating Scale [NRS]), ranging from 0 to 10 and as intensity categories (mild [NRS 1–3], moderate [NRS 4–6], and severe [NRS 7–10]), to provide complementary quantitative and clinically meaningful information, pain quality (pulsating, pressing/tightening), localization at the onset of migraine attacks (unilateral/bilateral), pain localization and diffusion, accompanying symptoms (nausea and/or vomiting, photophobia and/or phonophobia, ictal and interictal allodynia, dizziness, motor agitation, conjunctival redness, nasal congestion, eyelid edema, lacrimation, miosis/ptosis, sensation of ear fullness, sweating), aggravation by, or avoidance of, routine physical activity;
– Common triggers, including sexual activity, alcohol use, menstruation, physical exertion, relaxation, stress, sleep deprivation, cough or defecation and weather variations;
– Preventive and acute treatments ongoing at the index visit or previously prescribed. Preventive therapies included antidepressants, antiseizure medications, beta‐blockers, sartans, calcium‐channel blockers, selective serotonin reuptake inhibitors (SSRIs), serotonin and norepinephrine reuptake inhibitors (SNRIs), onabotulinumtoxinA (BoNT‐A), nerve blocks, anti‐CGRP monoclonal antibodies (mAbs), and supplements. Acute treatments included nonsteroidal anti‐inflammatory drugs (NSAIDs), triptans, ergot derivatives, opioids, paracetamol, and combination analgesics.
– Comorbid conditions including neurological, cardiovascular, psychiatric, endocrine‐metabolic, gastrointestinal, immunological, and oncological domains based on the presence of well‐defined diagnoses or, alternatively, on the intake of specific drugs:
– Lifestyle variables included: tobacco use, alcohol intake, caffeine consumption, recreational drug use, and sleep quality;
– Structured family history for headache, presence of aura, cardiovascular diseases, and psychiatric conditions.

### 2.3 Statistical Analysis

Given the descriptive nature of the study, no formal sample size calculation was performed and all available entries were included. Analyses were conducted using complete‐case data without imputation; the number of available observations for each variable is reported throughout. Data distribution was assessed visually and with the Shapiro–Wilk test. Nonparametric tests were applied when appropriate. Analyses concerning headache diagnoses were performed on the entire registry cohort, whereas analyses of migraine characteristics were restricted to patients with a primary diagnosis of migraine (migraine with aura [MA], migraine without aura [MWA], chronic migraine [CM], or migraine complications according to the ICHD‐3 classification). Patients with CM and concomitant MOH were included within the CM group, while MOH was analyzed separately as an additional diagnosis.

Categorical variables were presented as frequencies (*n*, %) and compared across age groups using *χ* ^2^ tests. For each contingency table, the Bonferroni‐adjusted significance level was calculated by dividing *α* = 0.05 by the total number of adjusted standardized residuals examined (i.e., the number of cells in the contingency table), and the corresponding two‐sided critical *z*‐value was used to determine statistical significance. Continuous variables were analyzed using Kruskal–Wallis analysis of variance (ANOVA), followed by Bonferroni‐corrected Dunn tests for pairwise comparisons. Given the clinical relevance of the oldest‐old population in geriatric research, an additional descriptive subgroup analysis was performed in patients aged > 70 years [24, 25, 26, 27]. Results are presented as mean ± standard deviation (SD), unless otherwise stated. Statistical significance was set at a two‐sided *p* < 0.05, with correction for multiple comparisons where appropriate. Analyses were performed using Jamovi software (version 2.6.13; Sydney, Australia).

## 3 Results

A total of 4081 patient records were retrieved. After excluding individuals aged < 18 years (*n* = 71) and records with missing data (*n* = 533), the final sample included 3477 patients (Figure 1). Of these, 293 patients (8.4%) were included in our previously published single‐center cohort [4]. Mean age in the whole sample was 46.7 ± 13.9 years, and 2715 (78.1%) were female. Patients were stratified into elderly (*n* = 644, 66.4 ± 6.1 years), middle‐aged (*n* = 1285, 52.2 ± 3.9 years), adults (*n* = 990, 38.6 ± 4.3 years), and young groups (*n* = 558, 25.4 ± 3.2 years).

**FIGURE 1:**
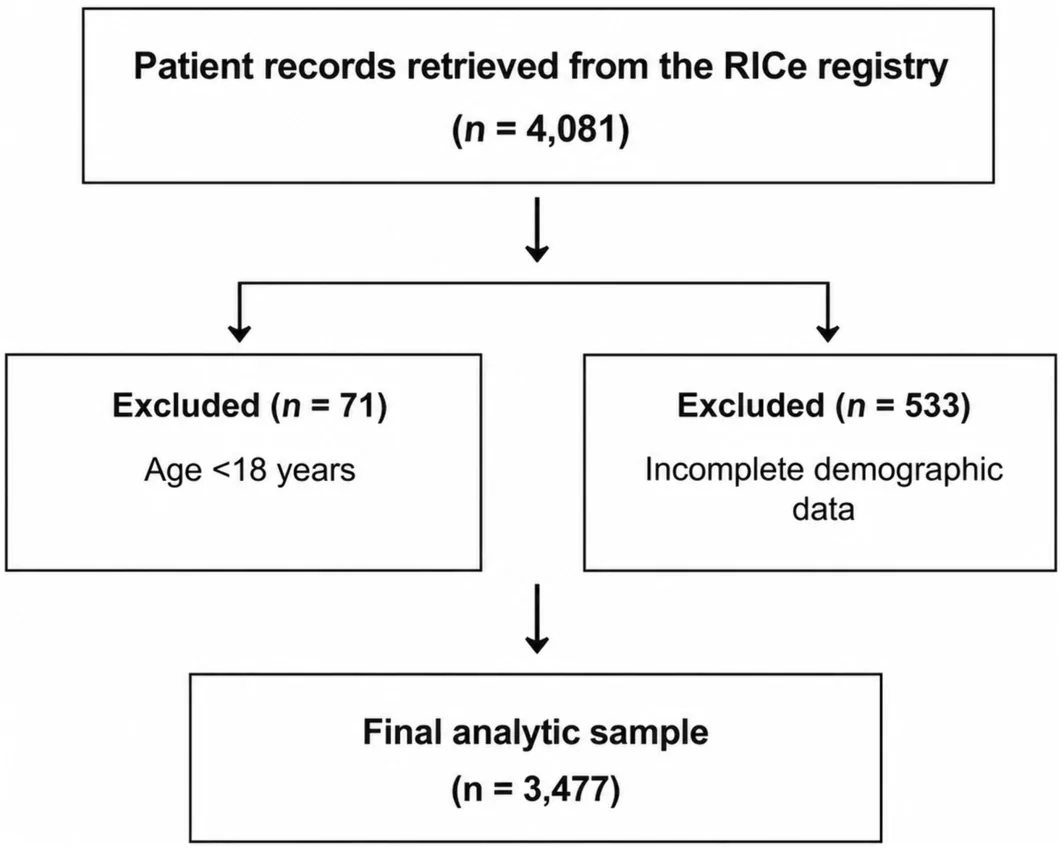
Flowchart of patient selection. Of 4081 patient records retrieved from the Italian Registry for Headache Disorders (RICe), 71 individuals aged < 18 years and 533 patients with incomplete demographic data were excluded, resulting in a final analytic sample of 3477 patients.

### 3.1 Headache Diagnosis According to ICHD‐3

Among the final cohort, 3013/3477 patients (86.7%) had migraine and 464 (13.3%) other headache diagnoses. Among non‐migraine diagnoses, episodic tension‐type headache (eTTH; 15.1%), trigeminal autonomic cephalalgias (TACs; 19.2%), including cluster headache, paroxysmal hemicrania, short‐lasting unilateral neuralgiform headache attacks and hemicrania continua, and chronic tension‐type headache (cTTH; 6.3%) were the most frequent. The distribution of non‐migraine ICHD‐3 diagnoses differed across age groups (*χ* ^2^(24) = 62.3, *p* < 0.001), with trigeminal neuralgias more represented in the elderly and secondary headaches in middle‐aged patients (Figure 2, Table S1).

**FIGURE 2:**
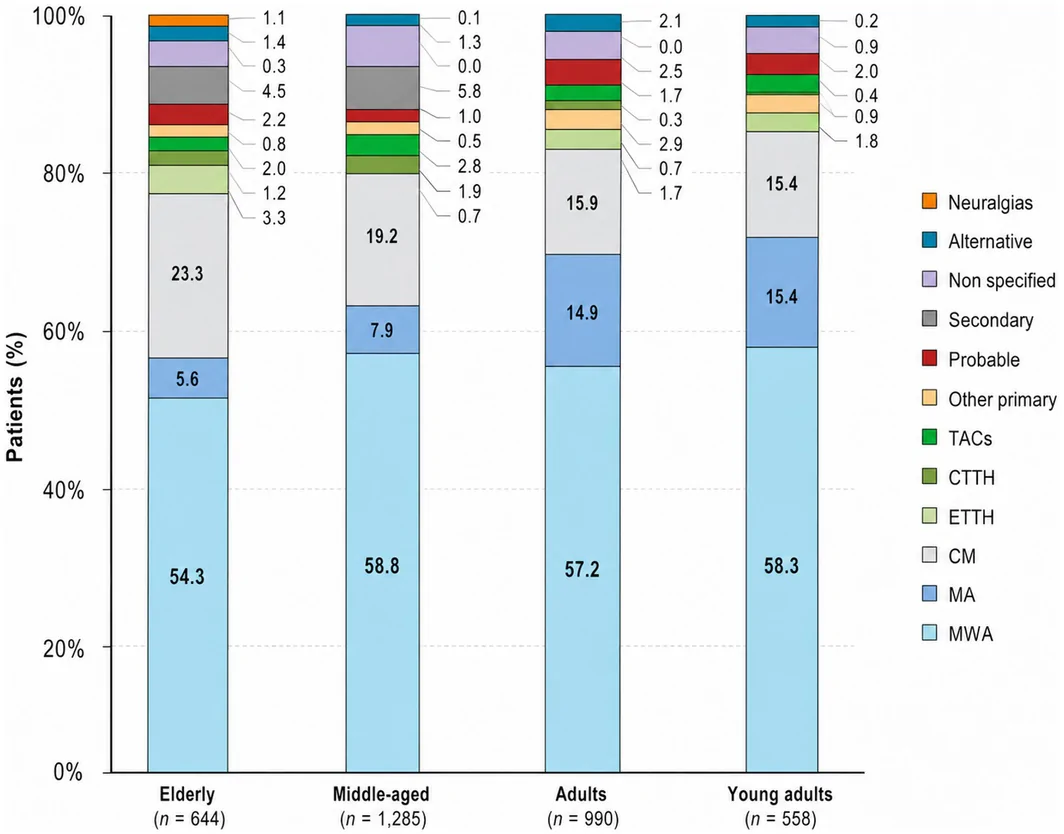
Percentage of patients with specific diagnoses according to the criteria of the International Headache Society across age subgroups, defined as follows: Elderly (≥ 60 years), middle‐aged (46–59 years), adults (31–45 years), and young adults (18–30 years). Other primary refers to headache disorders other than MWA, MA, TTH and TACS. Secondary refers to headache disorders secondary to other medical conditions. Probable includes probable migraine, probable TTH, probable TAC and other probable headache disorders. Alternative included diagnoses performed with alternative criteria included in the Appendix of the ICHD‐3. CM, chronic migraine; CTTH, chronic tension‐type headache; ETTH, episodic tension‐type headache; MA, migraine with aura; MWA, migraine without aura; TACs, trigeminal autonomic cephalalgias.

### 3.2 Migraine Across Age Groups

#### 3.2.1 Migraine Diagnosis

Among migraine patients (*n* = 3013), 536 (17.8%) were elderly (66.0 ± 5.9 years), 1104 (36.6%) middle‐aged (52.3 ± 3.9 years), 872 (28.9%) adults (38.6 ± 4.3 years), and 501 (16.6%) young individuals (25.4 ± 3.2 years) (Figure 3, Tables 1 and 2, S2). Sex distribution did not differ across groups (*n* = 3013, *χ* ^2^(3) = 1.80, *p* = 0.61). Mean age at migraine onset (*n* = 2003) differed significantly (H(3) = 120, *p* < 0.001; elderly: 22.9 ± 14.2 years, middle‐aged: 20.8 ± 14.3 years, adults: 17.5 ± 8.17 years, young: 14.0 ± 5.15 years), with significant differences between all groups except elderly and middle‐aged patients (*p* = 0.14).

**FIGURE 3:**
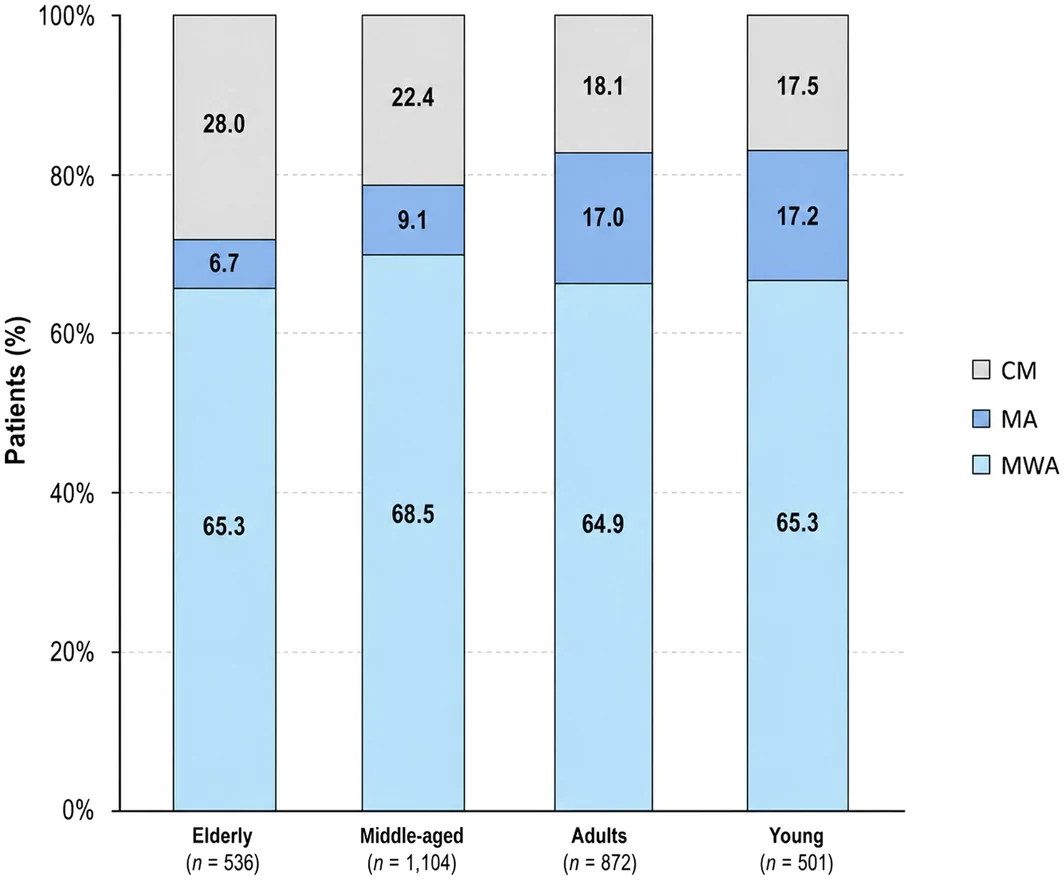
Percentage of patients with migraine without aura (MWA), migraine with aura (MA), and chronic migraine (CM) across age subgroups, defined as follows: Elderly (≥ 60 years), middle‐aged (46–59 years), adults (31–45 years), and young adults (18–30 years). The frequency of diagnoses varied across age groups [*χ* ^2^(6) = 71.5, *p* < 0.001]. CM was more frequent in older adults (28.0%, *z* = 4.18), whereas MA was less frequent in older and middle‐aged adults (6.7%, *z* = −4.35 and 9.1%, *z* = −4.02, respectively) and more frequent in young adults and adults (17.2%, *z* = 3.62 and 17.0%, *z* = 4.97, respectively). The Bonferroni‐corrected significance threshold was |*z*| ≥ 2.87. Only 2 patients (not shown in the figure) received a diagnosis of migraine complication, one within the young, the other within the adult group. CM included patients with and without an additional diagnosis of medication‐overuse headache (MOH).

**TABLE 1:** Categorical clinical characteristics of patients with migraine, including migraine without aura (MWA), migraine with aura (MA), chronic migraine (CM), and migraine complications according to age group.

	Elderly (*n* = 536)	Middle‐aged (*n* = 1104)	Adults (*n* = 872)	Young (*n* = 501)	Total (*n* = 3013)	Adjusted standardized residuals (*z*)
Sex (F) (*N*: 3013)	439 (81.9)	893 (80.9)	691 (79.2)	407 (81.2)	2430 (80.7)	0.81; 0.25; −1.25; 0.36
Headache FH (Y) (*N*: 235)	27 (65.9)	48 (63.2)	38 (55.9)	33 (66.0)	146 (62.1)	0.54; 0.23; −1.26; 0.64
Migraine with aura FH (Y) (*n* = 234)	1 (2.4)	1 (1.3)	1 (1.5)	2 (4.1)	5 (2.1)	0.15; −0.60; −0.45; 1.06
Pain laterality (bilateral) (*N*: 666)	30 (27.0)	67 (29.0)	48 (24.6)	50 (38.8)	195 (29.3)	−0.57; −0.11; −1.70; 2.64
Pain intensity (severe) (*N*: 666)	36 (32.1)	111 (47.2)	94 (48.7)	51 (40.5)	292 (43.8)	**−2.74;** 1.30; 1.61; −0.85
Pain quality (pulsating) (*N*: 1642)	167 (60.9)	359 (60.8)	305 (63.5)	201 (67.4)	1032 (62.9)	−0.71; −1.26; 0.37; 1.82
Pain localization (*N*: 243)						
Temporal/frontal	17 (40.5)/19 (45.2)	38 (49.4)/44 (57.1)	32 (43.8)/33 (45.2)	24 (47.1)/29 (56.9)	111 (45.7)/125 (51.4)	−0.74; 0.78; −0.38; 0.22/−0.88; 1.21; −1.27; 0.87
Occipital/parietal	5 (11.9)/7 (16.7)	15 (19.5)/17 (22.1)	13 (17.8)/12 (16.4)	10 (19.6)/6 (11.8)	43 (17.7)/42 (17.3)	−1.08; 0.50; 0.03; 0.40/−0.12; 1.35; −0.23; −1.17
Pain diffusion (*N*: 243)						
Temporal/frontal	17 (40.5)/20 (47.6)	36 (46.8)/28 (36.4)	34 (46.6)/32 (43.8)	17 (33.3)/21 (41.2)	104 (42.8)/101 (41.6)	−0.33; 0.85; 0.78; −1.54/0.88; −1.12; 0.47; −0.06
Occipital/parietal	16 (38.1)/14 (33.3)	27 (35.1)/24 (31.4)	12 (16.4)/19 (26.0)	13 (25.5)/12 (23.5)	68 (28.0)/69 (28.4)	1.61; 1.67; −2.63; −0.45/0.78; 0.65; −0.54; −0.87

*Note:* Age groups were defined as follows: elderly (≥ 60 years), middle‐aged (46–59 years), adults (31–45 years), and young adults (18–30 years). We excluded “probable migraine” or diagnoses performed with alternative criteria of the Appendix of the ICHD‐3. Data are presented as number of subjects, with percentages reported in parentheses. The total number of available observations for each variable is also reported in parentheses. Group differences were assessed using the *χ* ^2^ test. Post hoc comparisons were performed using adjusted standardized residuals with Bonferroni correction. The Bonferroni‐adjusted significance level was calculated by dividing *α* = 0.05 by the total number of adjusted standardized residuals examined (i.e., the number of cells in the contingency table), and the corresponding two‐sided critical *z*‐value was used to determine statistical significance. Adjusted standardized residuals (*z*) are reported in the following order: older adults, middle‐aged adults, adults, and young adults. Values exceeding the Bonferroni‐corrected significance threshold are shown in bold.

Abbreviations: FH, family history; Y, yes.

**TABLE 2:** Prodromal and accompanying symptoms in patients with migraine without aura (MWA), migraine with aura (MA), chronic migraine (CM), and migraine complications according to age group.

	Elderly (*n* = 536)	Middle‐aged (*n* = 1104)	Adults (*n* = 872)	Young (*n* = 501)	Total (*n* = 3013)	Adjusted standardized residuals (*z*)
Aura symptoms (Y) (*N*: 2116)	60 (17.3)	161 (21.4)	211 (32.7)	105 (28.3)	537 (25.4)	**−3.79; −3.11; 5.10**; 1.43
Nausea and/or vomiting (Y) (*N*: 2428)	172 (41.2)	362 (41.5)	297 (41.0)	176 (42.4)	1007 (41.5)	−0.10; 0.03; −0.30; 0.43
Photophobia and phonophobia (Y) (*N*: 2429)	287 (69.0)	632 (72.5)	519 (71.7)	290 (69.5)	1728 (71.1)	−1.06; 1.09; 0.39; −0.79
Dizziness (Y) (*N*: 482)	16 (19.3)	31 (18.9)	23 (16.8)	18 (18.4)	88 (18.3)	0.26; 0.26; −0.53; 0.03
Motor agitation (Y) (*N*: 659)	4 (3.6)	9 (3.9)	4 (2.1)	4 (3.2)	21 (3.2)	0.29; 0.75; −1.03; 0.01
Conjunctival redness/eyelid edema (Y) (*N*: 658)	1 (0.9)/3 (2.7)	9 (3.9)/9 (3.9)	6 (3.1)/6 (3.1)	4 (3.2)/4 (3.2)	20 (3.0)/22 (3.3)	−1.43; 0.93; 0.10; 0.12
Nasal congestion (Y) (*N*: 657)	3 (2.7)	12 (5.2)	8 (4.2)	3 (2.4)	26 (4.0)	−0.73; 1.20; 0.20; −0.99
Lacrimation/miosis or ptosis (Y) (*N*: 659)	1 (0.9)/0 (0.0)	25 (10.8)/8 (3.5)	14 (7.3)/5 (2.6)	9 (7.2)/4 (3.2)	49 (7.4)/17 (2.6)	**−2.86**; 2.41; −0.09; −0.11
Sensation of ear fullness/sweating (Y) (*N*: 657)	0 (0.0)/3 (2.7)	0 (0.0)/6 (2.6)	1 (0.5)/5 (2.6)	0 (0.0)/2 (1.6)	1 (0.2)/16 (2.4)	−1.19; −0.37; 0.81; 0.65
Allodynia (Y) (*N*: 657)	27 (24.5)	50 (21.6)	37 (19.4)	33 (26.4)	147 (22.4)	0.60; −0.33; −1.18; 1.20

*Note:* Age groups were defined as follows: elderly (≥ 60 years), middle‐aged (46–59 years), adults (31–45 years), and young adults (18–30 years). We excluded “probable migraine” or diagnoses performed with alternative criteria of the Appendix of the ICHD‐3. Data are presented as number of subjects, with percentages reported in parentheses. The total number of available observations for each variable is also reported in parentheses. Group differences were assessed using the *χ* ^2^ test. Post hoc comparisons were performed using adjusted standardized residuals with Bonferroni correction. The Bonferroni‐adjusted significance level was calculated by dividing *α* = 0.05 by the total number of adjusted standardized residuals examined (i.e., the number of cells in the contingency table), and the corresponding two‐sided critical *z*‐value was used to determine statistical significance. Adjusted standardized residuals (*z*) are reported in the following order: older adults, middle‐aged adults, adults, and young adults. Values exceeding the Bonferroni‐corrected significance threshold are shown in bold. The variable “Aura symptoms” refers to the presence of aura manifestations irrespective of the diagnostic classification.

Abbreviation: Y, yes.

Headache diagnosis varied among groups (*n* = 3013, *χ* ^2^(6) = 71.5, *p* < 0.001). CM was more frequent in the elderly, whereas MA was more common in young and adult patients (Figure 3, Table S2). To further assess whether the association between age and CM was independent of potential confounders, we performed an exploratory multivariable logistic regression including age group (elderly vs. < 60 years), sex, and age at migraine onset. Elderly individuals remained independently associated with CM (OR 1.88, 95% CI 1.45–2.44; *p* < 0.001), whereas neither sex (OR 1.14, 95% CI 0.87–1.49; *p* = 0.346) nor age at migraine onset (OR 1.00, 95% CI 0.99–1.01; *p* = 0.974) showed an independent association.

Aura was less frequently reported in elderly and middle‐aged individuals (*n* = 2116, *χ* ^2^(3) = 38.0, *p* < 0.001) (Table 2).

An additional diagnosis of MOH differed across age groups (*n* = 1420, *χ* ^2^(3) = 37.1, *p* < 0.001), being more frequent in the elderly and less common in young individuals (Figure 4). Concerning the type of overused medications, the use of combinations of different medications, corresponding to ICHD‐3 Category 8.2.6, differed across age groups (*χ* ^2^(3) = 9.63, *p* = 0.022), with a numerically higher frequency in older adults (*z* = 2.45), although this post hoc difference did not remain significant after Bonferroni correction (Table 3). Among patients with CM (*n* = 641), MOH differed across age groups (*χ* ^2^(3) = 8.39, *p* = 0.039), being slightly less frequent in young individuals (*n* = 13/38, *z* = −2.71) and tending to be more frequent in the elderly (*n* = 47/76, *z* = 1.45).

**FIGURE 4:**
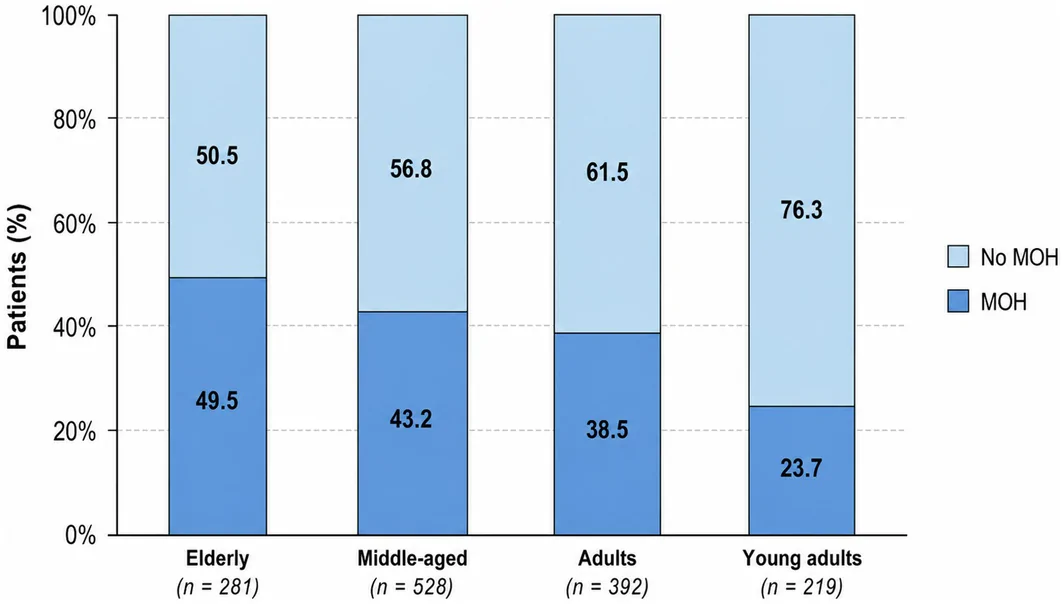
Percentage of migraine patients across the age groups with and without an additional diagnosis of Medication‐Overuse Headache (MOH). Age groups were defined as follows: Elderly (≥ 60 years), middle‐aged (46–59 years), adults (31–45 years), and young adults (18–30 years). The occurrence of an additional diagnosis of MOH (*n* = 1420) differed between age groups (*χ* ^2^(3) = 37.1, *p* < 0.001), as it was more frequently observed in the elderly (139 out of 281, *z* = 3.56) and less frequent in the young group (52 out of 219, *z* = −5.38) (corrected threshold: |*z*| ≥ 2.73).

**TABLE 3:** Acute symptomatic treatments in patients with migraine without aura (MWA), migraine with aura (MA), chronic migraine (CM), and migraine complications according to age group.

	Elderly (*n* = 160)	Middle‐aged (*n* = 300)	Adults (*n* = 212)	Young (*n* = 129)	Total (*n* = 801)	Adjusted standardized residuals (z)
NSAIDs (Y) (*N*: 801)	65 (40.6)	107 (35.7)	93 (43.9)	56 (43.4)	321 (40.1)	0.16; −1.97; 1.31; 0.84
Triptans (Y) (*N*: 801)	50 (31.3)	111 (37.0)	89 (42.0)	46 (35.7)	296 (37.0)	−1.67; 0.02; 1.77; −0.33
Ergot derivatives (*N*: 801)	1 (0.6)	1 (0.3)	1 (0.5)	0 (0)	3 (0.4)	0.58; −0.15; 0.27; −0.76
Paracetamol (*N*: 801)	20 (12.5)	33 (11.0)	30 (14.2)	20 (15.5)	103 (12.9)	−0.15; −1.22; 0.66; 0.98
Combination of symptomatic drugs (*N*: 801)	26 (16.3)	36 (12.0)	15 (7.1)	10 (7.8)	87 (10.9)	2.45; 0.80; −2.07; −1.24

*Note:* Age groups were defined as follows: elderly (≥ 60 years), middle‐aged (46–59 years), adults (31–45 years), and young adults (18–30 years). We excluded “probable migraine” or diagnoses performed with alternative criteria of the Appendix of the ICHD‐3. Data are presented as number of subjects, with percentages reported in parentheses. The total number of available observations for each variable is also reported in parentheses. Group differences were assessed using the *χ* ^2^ test. Post hoc comparisons were performed using adjusted standardized residuals with Bonferroni correction. The Bonferroni‐adjusted significance level was calculated by dividing *α* = 0.05 by the total number of adjusted standardized residuals examined (i.e., the number of cells in the contingency table), and the corresponding two‐sided critical *z*‐value was used to determine statistical significance. Adjusted standardized residuals (*z*) are reported in the following order: older adults, middle‐aged adults, adults, and young adults. Values exceeding the Bonferroni‐corrected significance threshold are shown in bold.

Abbreviations: NSAIDs, nonsteroidal anti‐inflammatory drugs; Y, yes.

#### 3.2.2 Migraine Frequency and Other Features

The elderly group attending headache centers reported higher monthly migraine days (MMDs) than young and adult groups (*n* = 1814, *H*(3) = 19.8, *p* < 0.001; *p* < 0.001 and *p* = 0.002, respectively) (Figure 5A).

**FIGURE 5:**
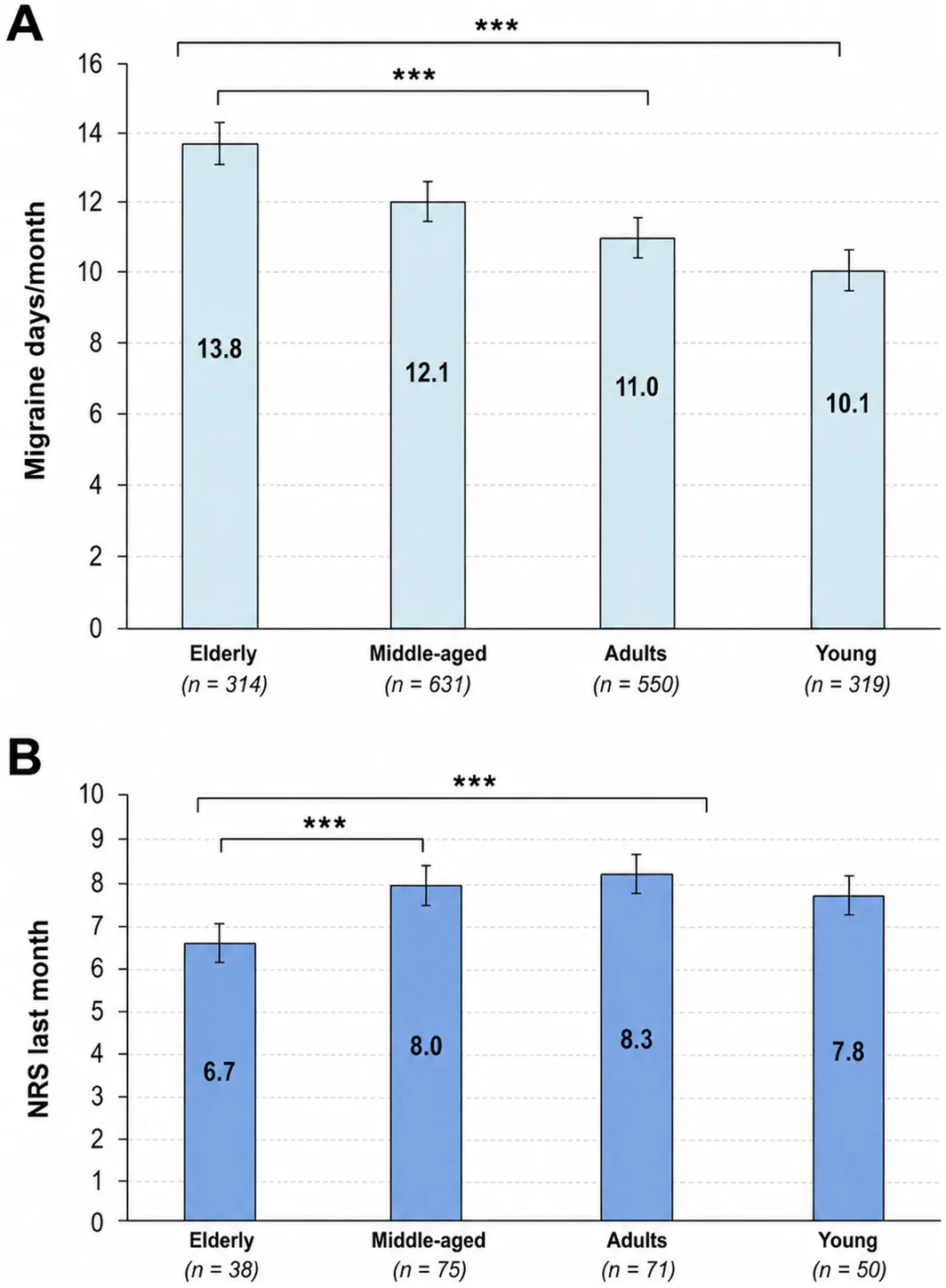
Monthly migraine days (A) and migraine intensity (B) across age groups. Elderly (≥ 60 years), middle‐aged (46–59 years), adults (31–45 years), and young adults (18–30 years). Migraine intensity was assessed using the Numeric Rating Scale (NRS). Bars represent mean values and error bars indicate the standard error of the mean (SEM). Older adults reported higher monthly migraine days than adults and young individuals (*H*(3) = 19.8, *p* < 0.001), whereas migraine intensity was lower in the elderly compared with adult and middle‐aged groups (*H*(3) = 17.3, *p* < 0.001). Significant pairwise comparisons are indicated in the figure.

Migraine intensity also differed across groups (*n* = 234, *H*(3) = 17.3, *p* < 0.001), with lower mean NRS scores in the elderly than in adult (*p* < 0.001) and middle‐aged patients (*p* = 0.003) (Figure 5B). Consistently, the proportion of patients reporting severe attacks (*n* = 666) was significantly lower in the elderly group (36/112 elderly, *χ* ^2^(3) = 9.76, *p* = 0.021) (Table 1).

No other differences emerged in migraine features or accompanying symptoms, except for lacrimation, which was rare and less frequent in the elderly (*n* = 659, *χ* ^2^(3) = 10.6, *p* = 0.014) (Table 1). Allodynia did not differ across groups (*n* = 657, *χ* ^2^(3) = 2.53, *p* = 0.47) (Table 2).

Regarding migraine triggers, physical activity (*n* = 242, *χ* ^2^(3) = 14.0, *p* = 0.003) and stress (*n* = 242, *χ* ^2^(3) = 10.7, *p* = 0.013) were more frequently reported in the elderly than in adults (Table S3).

Comorbidity data were available for a minority of patients (*n* = 235, Table S3). Arterial hypertension, endocrine‐metabolic disorders, anxiety, hypercholesterolemia, and smoking history were more frequent in the elderly (all *p* < 0.01). Hypothyroidism was more frequent in middle‐aged patients (*χ* ^2^(3) = 11.5, *p* = 0.009). Previous non‐cerebral neoplasms were slightly more frequent in elderly and middle‐aged groups, although this finding did not remain significant after correction (*χ* ^2^(3) = 17.2, *p* = 0.008).

Data on preventive treatments were available for 243 migraine patients (Tables 4 and 5). Regarding previous preventive treatments, beta‐blockers (*χ* ^2^(3) = 14.2, *p* = 0.003, *z* = 3.71) and botulinum toxin (*χ* ^2^(3) = 12.8, *p* = 0.005, *z* = 3.49) were more frequently used in the elderly (Table 4). Lack of efficacy was the main reason for discontinuation, whereas adverse effects were rare. Regarding ongoing preventive treatments, the use of BoNT‐A was slightly more frequent in elderly patients (*χ* ^2^(3) = 7.88, *p* = 0.04); however, this difference did not remain statistically significant after correction for multiple comparisons (Table 5). No age‐group differences emerged for anti‐CGRP monoclonal antibody prescription (*n* = 241; *χ* ^2^(3) = 3.13, *p* = 0.37), although galcanezumab was more frequently prescribed in adults (*χ* ^2^(3) = 9.4, *p* = 0.02, *z* = 2.47).

**TABLE 4:** Previous preventive treatments in patients with migraine without aura (MWA), migraine with aura (MA), chronic migraine (CM), and migraine complications according to age group.

	Elderly (*n* = 42)	Middle‐aged (*n* = 77)	Adults (*n* = 73)	Young (*n* = 51)	Total (*n* = 243)	Adjusted standardized residuals (z)
Amitriptyline (Y) (*N*: 243)	20 (47.6)	33 (42.9)	26 (35.6)	18 (35.3)	97 (39.9)	1.12; 0.64; −0.90; −0.76
Topiramate (Y) (*N*: 243)	17 (40.5)	28 (36.4)	21 (28.8)	10 (19.6)	76 (31.3)	1.41; 1.17; −0.55; −2.02
Valproate (Y) (*N*: 243)	7 (16.7)	6 (7.8)	4 (5.5)	2 (3.9)	19 (7.8)	2.35; −0.01; −0.89; −1.17
Lamotrigine (Y) (*N*: 243)	2 (4.8)	6 (7.8)	2 (2.7)	3 (5.9)	13 (5.3)	−0.19; 1.15; −1.18; 0.19
Gabapentinoid (*N*: 243)	7 (16.7)	10 (13.0)	4 (5.5)	3 (5.9)	24 (9.9)	1.62; 1.11; −1.51; −1.08
Beta blockers (*N*: 243)	17 (40.5)	11 (14.3)	13 (17.8)	7 (13.7)	48 (19.8)	**3.71;** −1.46; −0.50; −1.22
Sartans (*N*: 243)	1 (2.4)	1 (1.3)	0 (0.0)	1 (2.0)	3 (1.2)	0.74; 0.06; −1.14; 0.53
CCBs (*N*: 243)	14 (33.3)	25 (32.5)	15 (20.5)	8 (15.7)	62 (25.5)	1.28; 1.69; −1.16; −1.81
SSRI (*N*: 243)	5 (11.9)	7 (9.1)	5 (6.8)	1 (2.0)	18 (7.4)	1.22; 0.68; −0.22; −1.67
SNRI (*N*: 243)	4 (9.5)	7 (9.1)	0 (0.0)	1 (2.0)	12 (4.9)	1.51; 2.03; −2.33; −1.10
OnabotulinumtoxinA (BoNT‐A) (*N*: 243)	10 (23.8)	5 (6.5)	6 (8.2)	2 (3.9)	23 (9.5)	**3.49**; −1.08; −0.44; −1.52
Nerve block (*N*: 243)	2 (4.8)	0 (0.0)	1 (1.4)	0 (0.0)	3 (1.2)	2.28; −1.19; 0.13; −0.90
Magnesium (*N*: 243)	2 (4.8)	1 (1.3)	4 (5.5)	3 (5.9)	10 (4.1)	0.23; −1.51; 0.70; 0.72
Anti‐CGRP mAbs (*N*: 243)	6 (14.3)	5 (6.5)	9 (12.3)	4 (7.8)	24 (9.9)	1.05; −1.20; 0.84; −0.55

*Note:* Age groups were defined as follows: elderly (≥ 60 years), middle‐aged (46–59 years), adults (31–45 years), and young adults (18–30 years). We excluded “probable migraine” or diagnoses performed with alternative criteria of the Appendix of the ICHD‐3. Data are presented as number of subjects, with percentages reported in parentheses. The total number of available observations for each variable is also reported in parentheses. Group differences were assessed using the *χ* ^2^ test. Post hoc comparisons were performed using adjusted standardized residuals with Bonferroni correction. The Bonferroni‐adjusted significance level was calculated by dividing *α* = 0.05 by the total number of adjusted standardized residuals examined (i.e., the number of cells in the contingency table), and the corresponding two‐sided critical *z*‐value was used to determine statistical significance. Adjusted standardized residuals (*z*) are reported in the following order: older adults, middle‐aged adults, adults, and young adults. Values exceeding the Bonferroni‐corrected significance threshold are shown in bold.

Abbreviations: anti‐CGRP mAbs, monoclonal antibodies targeting the calcitonin gene‐related peptide pathway; CCBs, calcium‐channel blockers; SNRIs, serotonin and norepinephrine reuptake inhibitors; SSRIs, selective serotonin reuptake inhibitors; Y, yes.

**TABLE 5:** Ongoing preventive treatments in patients with migraine without aura (MWA), migraine with aura (MA), chronic migraine (CM), and migraine complications according to age group.

	Elderly (*n* = 42)	Middle‐aged (*n* = 77)	Adults (*n* = 73)	Young (*n* = 51)	Total (*n* = 243)	Adjusted standardized residuals (*z*)
Amitriptyline (Y) (*N*: 243)	12 (28.6)	21 (27.3)	29 (39.7)	16 (31.4)	78 (32.1)	−0.54; −1.10; 1.67; −0.13
Topiramate (Y) (*N*: 243)	0 (0.0)	8 (10.4)	10 (13.7)	8 (15.7)	26 (10.7)	−2.47; −0.11; 0.99; 1.30
Valproate (Y) (N: 243)	0 (0.0)	1 (1.3)	0 (0.0)	0 (0.0)	1 (0.4)	−0.46; 1.47; −0.66; −0.52
Lamotrigine (Y) (*N*: 243)	1 (2.4)	3 (3.9)	1 (1.4)	0 (0.0)	5 (2.1)	0.16; 1.37; −0.50; −1.16
Gabapentinoid (*N*: 243)	4 (9.5)	4 (5.2)	3 (4.1)	0 (0.0)	11 (4.5)	1.71; 0.34; −0.21; −1.75
Beta blockers (*N*: 243)	3 (7.1)	13 (16.9)	8 (11.0)	3 (5.9)	27 (11.1)	−0.90; 1.95; −0.05; −1.34
Sartans (*N*: 243)	0 (0.0)	0 (0.0)	1 (1.4)	2 (3.9)	3 (1.2)	−0.80; −1.19; 0.13; 1.95
CCBs (*N*: 243)	0 (0.0)	1 (1.3)	3 (4.1)	2 (3.9)	6 (2.5)	−1.13; −0.80; 1.08; 0.75
SSRI (*N*: 243)	1 (2.4)	4 (5.2)	5 (6.8)	3 (5.9)	13 (5.3)	−0.94; −0.07; 0.68; 0.19
SNRI (*N*: 243)	1 (2.4)	2 (2.6)	1 (1.4)	0 (0.0)	4 (1.6)	0.41; 0.79; −0.22; −1.04
OnabotulinumtoxinA (BoNT‐A) (*N*: 243)	3 (7.1)	4 (5.2)	0 (0.0)	0 (0.0)	7 (2.9)	1.82; 1.47; −1.76; −1.38
Nerve block (*N*: 243)	0 (0.0)	3 (3.9)	0 (0.0)	0 (0.0)	3 (1.2)	−0.80; 2.56; −1.14; −0.90
Magnesium (*N*: 243)	2 (4.8)	4 (5.2)	7 (9.6)	9 (17.6)	22 (9.1)	−1.07; −1.43; 0.19; 2.41
Anti‐CGRP mAbs (*N*: 241)	35 (83.3)	68 (89.5)	62 (84.9)	39 (78.0)	204 (84.6)	0.26; −1.41; −0.08; 1.46

*Note:* Age groups were defined as follows: elderly (≥ 60 years), middle‐aged (46–59 years), adults (31–45 years), and young adults (18–30 years). We excluded “probable migraine” or diagnoses performed with alternative criteria of the Appendix of the ICHD‐3. Data are presented as number of subjects, with percentages reported in parentheses. The total number of available observations for each variable is also reported in parentheses. Group differences were assessed using the *χ* ^2^ test. Post hoc comparisons were performed using adjusted standardized residuals with Bonferroni correction. The Bonferroni‐adjusted significance level was calculated by dividing *α* = 0.05 by the total number of adjusted standardized residuals examined (i.e., the number of cells in the contingency table), and the corresponding two‐sided critical *z*‐value was used to determine statistical significance. Adjusted standardized residuals (*z*) are reported in the following order: older adults, middle‐aged adults, adults, and young adults. Values exceeding the Bonferroni‐corrected significance threshold are shown in bold. anti‐CGRP mAbs, monoclonal antibodies targeting the calcitonin gene‐related peptide pathway; CCBs, calcium‐channel blockers; MOH, medication‐overuse headache; SSRIs, selective serotonin reuptake inhibitors; SNRIs, serotonin and norepinephrine reuptake inhibitors; Y, yes. For anti‐CGRP mAbs, data were available for 241 patients (42 elderly, 76 middle‐aged, 73 adults, 50 young).

#### 3.2.3 Focus on > 70 Years Migraine Patients

To further characterize migraine in the oldest population attending headache centers, we analyzed patients aged > 70 years (*n* = 115; 77.4% female; mean age 75.1 ± 5.5 years; mean age at onset 22.7 ± 15.2 years). Migraine diagnoses mirrored those of the overall elderly group, with MWA as the most frequent diagnosis (67.0%), followed by CM (27.0%) and MA (6.1%). Almost half of the patients (47.5%) had an additional diagnosis of MOH.

Migraine frequency was high (18.1 ± 9.8 MMDs), whereas pain intensity remained relatively low (NRS 6.5 ± 1.9). Pain was bilateral in 35% of patients and most often pulsating (62.5%). Most patients reported photophobia and phonophobia (67%), and nearly half experienced nausea or vomiting (42.3%). Data on triggers, allodynia, and comorbidities were frequently unavailable.

## 4 Discussion

In this large multicenter registry‐based study of patients attending Italian headache centers, we examined age‐related differences in headache phenotypes, clinical characteristics, and treatment patterns, with a specific focus on elderly individuals with migraine. Compared with previous single center cohorts [4, 6, 13, 16, 28], the present dataset provides a broader and more representative overview of real‐world clinical practice, allowing a more robust evaluation of how migraine manifests across the lifespan. Focusing on the 3013 migraine diagnoses, the main findings related to the elderly group were: (i) a higher rate of CM and CM‐MOH diagnoses, with a tendency toward more frequent use of combinations of symptomatic treatments; (ii) a higher number of MMDs, lower pain intensity and fewer severe attacks; (iii) physical activity and stress as more common triggering factors; (iv) a greater use of beta‐blockers and BoNT‐A as preventive treatments, while no age‐related differences were observed in the prescription of anti‐CGRP monoclonal antibodies.

Although the RICe registry primarily includes patients with migraine, it also comprised a large subgroup of non‐migraine headache disorders (> 400 patients), exceeding the sample size of previous studies on older headache populations [4, 12, 28, 29]. Middle‐aged patients showed a higher frequency of secondary headaches, whereas elderly patients more frequently presented neuralgias. While this latter finding is consistent with previous observations in older headache populations [28], the overall distribution of non‐migraine headache diagnoses appeared more homogeneous than reported in monocentric studies [4, 6, 16]. Unlike previous monocentric reports describing a higher burden of TTH, particularly cTTH, among older adults [4, 30], no clear age‐related predominance emerged in the present multicenter cohort. However, these findings should be interpreted cautiously, as the non‐consecutive recruitment strategy and the absence of analyses on coexisting headache diagnoses (except for MOH) may have influenced diagnostic distribution.

### 4.1 Migraine Diagnosis

Migraine was confirmed as the predominant diagnosis (86.7%) across age groups, reflecting referral patterns to specialized headache centers and the recruitment characteristics of the RICe registry [31, 32, 33]. Among elderly individuals, CM was more frequent, whereas MA was less common, consistent with our previous Italian monocentric study [4] and other reports in older headache populations [12, 28]. The independent association between older age and CM was further confirmed by an exploratory multivariable logistic regression adjusted for sex and age at migraine onset, which yielded results consistent with the univariate analyses. Conversely, MA was more common in young and adult patients, in line with previous RICe analyses showing that patients with aura were younger and had an earlier disease onset [20].

The lower frequency of MA in the elderly contrasts, however, with reports suggesting that aura may become more frequent with advancing age [1, 13]. Previous evidence has also shown that late‐life onset migraine with aura is not uncommon and may occur without headache, particularly in older individuals [34]. Therefore, an under‐recognition of aura in our cohort cannot be excluded, and this aspect deserves further investigation.

### 4.2 Migraine Features Across Age Groups

Age at migraine onset progressively increased across age groups, with elderly patients reporting later onset than younger individuals. However, consistent with previous observations, most elderly individuals described a long‐standing disease course, suggesting that the high representation of CM in this subgroup may reflect progressive chronification over decades rather than late‐onset migraine [28, 35, 36, 37]. Even among elderly patients, the mean age at migraine onset remained well below 65 years, supporting the interpretation that migraine in this population generally reflects persistence and evolution of a pre‐existing disorder rather than de novo late‐life onset. In line with this, onset of primary headache disorders after the age of 65 is uncommon (< 5%) and should prompt evaluation for possible secondary causes [28].

Headache frequency was higher among elderly patients, consistent with the greater representation of CM in this group. In contrast, pain intensity, as measured by the NRS, was significantly lower, and fewer elderly individuals reported severe attacks, in agreement with recent findings [15]. A similar pattern was observed in the oldest subgroup (> 70 years) [24, 25, 26, 27], supporting age‐related modulation of nociceptive processing, potentially reflecting adaptive mechanisms associated with long‐standing disease and/or neurobiological changes in central pain processing [38, 39]. The relatively small sample size of this subgroup, however, may increase the risk of selection bias and limit the generalizability of our findings.

Core migraine features, including pain quality, localization, and diffusion, did not differ significantly across age groups, suggesting relative stability of the migraine phenotype throughout life. Likewise, nausea, vomiting, photophobia, and phonophobia were largely comparable, with lacrimation representing the only symptom less frequently reported among the elderly. This contrasts with our previous monocentric study [4], in which elderly patients showed fewer accompanying migraine symptoms overall. Similarly, allodynia frequency did not differ across age groups in the present cohort, contrary to our previous findings [4]. However, in the earlier study, allodynia was assessed using validated questionnaires rather than simple presence/absence reporting, which may partly explain this discrepancy.

Stress and physical activity were more frequently reported as migraine triggers among the elderly [6]. This finding may reflect increased vulnerability to internal and external stressors in later life and contributes to the persistence of migraine burden and its chronification in this population [35, 36, 37]. On the other hand, the greater reporting of physical activity as a trigger in elderly individuals may warrant further investigation to exclude secondary causes of exertional headache, which may become more relevant with age.

Concerning comorbidities, we observed slightly higher rates of psychiatric disorders, particularly anxiety, in elderly patients. Endocrine‐metabolic disorders, hypercholesterolemia, arterial hypertension, and previous smoking were also more frequent in older individuals, as expected. These findings reinforce the concept that migraine in older adults occurs within a complex comorbidity framework, thereby increasing therapeutic challenges [2, 8, 9, 12, 14]. The broader multicenter sampling likely enabled a more accurate capture of systemic conditions compared with the monocentric dataset.

### 4.3 Medication‐Overuse Headache and Treatment Patterns

A key finding of this study is the significantly higher representation of MOH among elderly patients, in agreement with monocentric cohorts [4, 12, 15]. A tendency toward more frequent use of combinations of acute medications (Category 8.2.6 of the ICHD‐3), including triptans, NSAIDs, paracetamol, ergot derivatives, and opioids, was also observed in older adults, suggesting polypharmacy and increased vulnerability to adverse events. Interestingly, triptan use was also observed among some patients aged > 60 years. Given historical concerns regarding cardiovascular safety in older populations [40], this finding may reflect evolving prescribing practices in clinically selected patients, although further studies are needed to clarify appropriateness and safety.

Higher headache frequency in the elderly may partly explain the greater use of acute symptomatic treatments, potentially contributing to the increased representation of CM and MOH. At the same time, a bidirectional relationship is likely, whereby chronification itself may further promote symptomatic medication use [35, 36, 37, 39]. In this context, lower attack intensity in elderly patients should not necessarily be interpreted as a marker of milder disease. Rather, it may reflect more prompt or extensive use of acute therapies, potentially attenuating pain severity, although this hypothesis warrants further investigation.

Although preventive migraine treatments were also reported among older adults [4, 41], missing information regarding previous or ongoing prophylactic therapies limits the interpretability and generalizability of our findings. Among preventive options, beta‐blockers and BoNT‐A were more frequently prescribed in elderly patients, likely reflecting their perceived tolerability and clinical familiarity in this age group [42, 43].

Regarding anti‐CGRP monoclonal antibodies, complete treatment information was available for only 42 elderly patients. No significant age‐related differences in prescribing patterns emerged. Although the limited number of treated elderly patients precludes definitive conclusions, these findings may indicate an increasing consideration of anti‐CGRP therapies as a therapeutic option also in later life within specialized headache practice [44].

Overall, our findings could be interpreted in the context of the greater comorbidity burden and frequent polypharmacotherapy observed in older adults. In this population, both acute and preventive migraine treatments require careful clinical consideration because of potential cardiovascular risks, drug–drug interactions, and tolerability issues. For example, frequent NSAID use may raise concerns regarding cardiovascular, renal, and gastrointestinal safety, whereas preventive options, including anti‐CGRP monoclonal antibodies, may require additional caution in selected patients with uncontrolled hypertension or complex vascular comorbidities. Therefore, individualized treatment strategies and careful monitoring remain particularly important in older adults with migraine [45].

### 4.4 Strengths and Limitations

Building upon our previous single‐center study [4], the present analysis is based on prospectively collected real‐world data from 3477 individuals recruited across 30 headache centers participating in the Italian RICe registry, substantially improving the representativeness and external validity of the findings. The multicenter design allowed us to determine whether the clinical characteristics previously observed in a single tertiary headache center could be confirmed across different geographical regions and clinical settings, thereby providing an external multicenter validation of our earlier observations. In addition, the present study includes a substantially larger migraine population (3013 vs. 2390 patients) and, most importantly, more than doubles the number of elderly patients with migraine (536 vs. 216 patients). This larger and more heterogeneous cohort enabled a more comprehensive characterization of migraine subtypes, medication‐overuse headache, treatment patterns, and age‐related clinical features encountered in routine clinical practice. Data were collected using a semi‐structured assessment performed by expert headache specialists, which enhances diagnostic accuracy and internal validity. Standardized ICHD‐3 diagnostic criteria improve reproducibility and comparability with previous studies. The inclusion of demographic features, disease characteristics, comorbidities, triggers, and treatment patterns enabled a comprehensive phenotypic characterization across age groups. The relatively large sample of older adults, often underrepresented in migraine research, further strengthens the clinical relevance of the findings.

However, several limitations must be also acknowledged. The cross‐sectional design precludes inference on temporal evolution or causal relationships, particularly regarding disease progression and chronification. Information on timing of first migraine diagnosis was unavailable. Although age at headache onset was recorded, we could not assess delays between symptom onset and diagnosis, nor their impact on disease progression or treatment patterns. This may be particularly relevant in older adults, in whom atypical or attenuated clinical presentations could contribute to under‐recognition or delayed referral. Future longitudinal studies are warranted to better characterize diagnostic pathways and their implications for migraine management across the lifespan. Moreover, restricting the migraine‐specific analyses to definite diagnoses may have resulted in some underrepresentation of atypical migraine presentations, particularly among elderly patients. Patients were recruited from specialized headache centers, resulting in a clinically selected population that may not reflect the broader spectrum of migraine in the general population. Therefore, these findings should be interpreted as representative of real‐world headache center populations rather than epidemiological estimates. Several variables showed substantial missingness, limiting statistical power and interpretability of subgroup analyses. Heterogeneity in data completeness across centers and variables may reflect differences in clinical practice or documentation, potentially affecting internal consistency. Furthermore, the availability of some variables reflected the progressive implementation of specific data fields within the registry. Missing data were not systematically concentrated within specific age groups or headache centers but primarily reflected differences in the availability of selected variables across the registry over time. Therefore, analyses of comorbidities, triggering factors, and treatment patterns should be considered exploratory and interpreted with caution because of the limited number of available observations. In addition, some variables (e.g., ergot derivatives, sartans, and nerve blocks) contained sparse cell counts, for which the chi‐square approximation may be less reliable. Although multiple comparisons were performed with correction procedures, residual risk of Type I error cannot be excluded. For analytical purposes, we considered only the primary headache diagnosis, in line with the previous Italian monocentric study [4]. While ensuring consistency and interpretability in a large multicenter dataset, this approach represents a simplification of clinical reality, as it does not account for concomitant headache diagnoses. A further limitation concerns potential selection bias. In older adults, migraine may lose its typical painful component, presenting instead with non‐painful manifestations such as vestibular symptoms or nausea. These atypical presentations are less likely to prompt referral to neurologists and, even more so, to specialized headache centers. Consequently, our cohort predominantly captures cases in which pain is a prominent symptom—the feature that most commonly drives patients to seek specialist evaluation. This may lead to an underrepresentation of non‐painful migraine presentations in the elderly, and should be considered when interpreting the findings, as it impacts the generalizability of our results and highlights the need for greater clinical awareness of these less typical manifestations. Although an exploratory multivariable analysis confirmed the independent association between older age and CM after adjustment for sex and age at migraine onset, residual confounding cannot be excluded. Center‐level variability, temporal trends in data collection, and other potential confounders were not specifically modeled. Finally, although the middle‐aged age group was selected to encompass the period during which most women undergo menopause, the present study was not designed to evaluate the independent effects of sex or menopausal status. Future studies specifically addressing hormonal status and menopause may further improve the understanding of age‐related changes in migraine phenotype.

## 5 Conclusion

Among patients attending specialized headache centers, elderly individuals with migraine more frequently presented chronic migraine and medication‐overuse headache, together with a pattern of frequent but overall less severe attacks, and a greater relevance of stress‐ and physical activity–related triggers. Rather than representing a distinct clinical entity, our findings suggest that migraine in older adults may be characterized by high headache frequency despite lower symptom intensity and greater therapeutic complexity. Given the registry‐based design and the selected clinical setting, these findings should be interpreted cautiously but may help inform age‐tailored diagnostic and therapeutic strategies. Prospective longitudinal studies are needed to better define disease trajectories and optimize migraine management in older adults.

## Author Contributions

**Giulia Paparella:** conceptualization, data curation, writing – original draft, formal analysis. **Luigi Francesco Iannone:** data curation, writing – review and editing. **Giulia Paparella:** investigation. **Grazia Sances:** investigation, writing – review and editing. **Roberto De Icco:** investigation, writing – review and editing. **Gloria Vaghi:** investigation, writing – review and editing. **Cristina Tassorelli:** investigation, writing – review and editing. **Andrea Burgalassi:** investigation, writing – review and editing. **Francesco De Cesaris:** investigation, writing – review and editing. **Alberto Chiarugi:** investigation, writing – review and editing. **Francesca Gabriele:** investigation, writing – review and editing. **Raffaele Ornello:** investigation, writing – review and editing. **Simona Sacco:** investigation, writing – review and editing. **Maria Pia Prudenzano:** investigation, writing – review and editing. **Martino Gentile:** investigation, writing – review and editing. **Damiano Paolicelli:** investigation, writing – review and editing. **Giorgia Dalla Volta:** investigation, writing – review and editing. **Marco Bolchini:** investigation, writing – review and editing. **Innocenzo Rainero:** investigation, writing – review and editing. **Elisa Rubino:** investigation, writing – review and editing. **Maria Albanese:** investigation, writing – review and editing. **Fabrizio Vernieri:** investigation, writing – review and editing. **Gianluca Coppola:** investigation, writing – review and editing. **Gianluca Avino:** investigation, writing – review and editing. **Edoardo Mampreso:** investigation, writing – review and editing. **Delfina Ferrandi:** investigation, writing – review and editing. **Marta Altieri:** investigation, writing – review and editing. **Stefania Battistini:** investigation, writing – review and editing. **Antonio Granato:** investigation, writing – review and editing. **Simona Guerzoni:** supervision. **Antonio Russo:** supervision, writing – review and editing. **Marina de Tommaso:** supervision, writing – review and editing.

## Funding

Giulia Paparella was supported by the Italian Ministry of Health (Current Research 2026). The funding body had no role in the study design, data collection, statistical analysis, interpretation of findings, manuscript preparation, or decision to submit the manuscript for publication.

## Ethics Statement

The local Ethics committee approved the study as part of the Registro Italiano Cefalee (RICe) study (Studio RICe, 14591_oss CEAVC Studio RICe, and subsequent amendments).

## Conflicts of Interest

L.F.I. received financial support, consulting/advisory board fees and meeting support from Teva, Eli Lilly, Lundbeck, Pfizer and AbbVie; he is Associate Editor for Frontiers in Neurology and Junior Editor of Cephalalgia and Cephalalgia Reports. G.S. received speaker/advisory board fees from Eli Lilly, Teva, Lundbeck, Organon and Pfizer, and travel grants from Teva and AbbVie. R.D.I. reports personal fees and non‐financial support from Eli Lilly, AbbVie, Pfizer, Lundbeck and Teva. M.P.P. received speaker/advisory board fees from AbbVie, Eli Lilly, Lundbeck, Organon, Pfizer and Teva, and meeting support from Teva. C.T. received research support from AbbVie and Novartis; advisory board/consulting fees from AbbVie, Dompé, Eli Lilly, Ipsen, Lundbeck, Medscape, Pfizer and Teva; speaker honoraria from AbbVie, Eli Lilly, Lundbeck, Pfizer and Teva; meeting support from AbbVie, Dompé, Eli Lilly, Ipsen, Lundbeck, Pfizer and Teva; served as PI in trials sponsored by AbbVie, Biohaven, Eli Lilly, Ipsen, Lundbeck, Pfizer and Teva; and received grants from the European Commission and the Italian Ministries of Health and University. R.O. reports personal fees from AbbVie, Bayer, Eli Lilly, Lundbeck, Novartis, Organon, Pfizer and Teva; he is Associate Editor for Arquivos de Neuro‐Psiquiatria and Editorial Board Member of The Journal of Headache and Pain and Confinia Cephalalgica. S.S. reports employment by the University of L'Aquila and compensation/consulting fees from Novartis, Novo Nordisk, Boehringer Ingelheim, Teva, Allergan, Pfizer Canada, Abbott Canada, Lundbeck, AstraZeneca and Eli Lilly. S.G. received fees/honoraria for advisory boards, speaker activities or clinical studies from Teva, Eli Lilly/Organon, Pfizer, Lundbeck, Angelini and AbbVie. D.P. received advisory board fees, speaker honoraria, travel support, research grants, consulting fees or clinical trial support from Alexion, Amgen, BMS‐Celgene, Biogen, Merck, Neuraxpharm, Novartis, Roche, Sandoz and Sanofi‐Genzyme. F.V. received research support from AbbVie, Angelini and Lundbeck; advisory board fees from AbbVie, Angelini, Eli Lilly, Lundbeck, Organon, Novartis, Pfizer and Teva; speaker honoraria from AbbVie, Eli Lilly, Lundbeck, Novartis, Pfizer and Teva; meeting support from AbbVie, Eli Lilly, Lundbeck, Pfizer and Teva; and served as PI in trials sponsored by AbbVie, Eli Lilly, Lundbeck, Pfizer and Teva. The other authors declare no conflicts of interest related to this manuscript.

## Supporting information


**Table S1:** Adjusted standardized residuals from the post hoc analysis of non‐migraine diagnoses across age groups. Residuals indicate the strength and direction of the association between each diagnosis and age group. Values greater than |3.20| are statistically significant after Bonferroni correction for multiple comparisons (*p* < 0.001389) and are shown in bold.
**Table S2:** Adjusted standardized residuals from the post hoc analysis of migraine diagnoses across age groups. The table shows the analysis performed on 3011 of the 3013 patients with migraine; the two patients with migraine complications (both diagnosed with status migrainosus, ICHD‐3 code 1.4.1) were excluded from this specific analysis. The contingency analysis included three mutually exclusive migraine diagnostic categories—migraine without aura (MWA), migraine with aura (MA), and chronic migraine (CM)—across the four age groups. The Bonferroni‐adjusted significance level was calculated by dividing *α* = 0.05 by the 12 adjusted standardized residuals examined (*α* = 0.00417), corresponding to a two‐sided critical value of |*z*| ≥ 2.87. Statistically significant residuals are shown in bold.
**Table S3:** Categorical clinical variables in patients with migraine without aura (MWA), migraine with aura (MA), chronic migraine (CM) and migraine complications. Age groups are defined as follows: elderly (≥ 60 years), middle‐aged (46–59 years), adults (31–45 years), and young (18–30 years). Data represent the number of subjects; percentages are reported in parentheses. The total number of subjects for each row is indicated in square brackets. Group differences were assessed using the *χ* ^2^ test. Post hoc comparisons were performed using adjusted standardized residuals and corrected for multiple comparisons with the Bonferroni method.

## Data Availability

The data that support the findings of this study are available on request from the corresponding author. The data are not publicly available due to privacy or ethical restrictions.
