# Supplementary material for: Migraine in Older Adults: Age‐Related Clinical Features From the Italian RICe Registry
Source: Eur J Neurol. 2026 Oct 14;33(10):e70761. doi: 10.1111/ene.70761 (PMC13664672; doi:10.1111/ene.70761)
Supplement: Supplementary file 1 [file ENE-33-e70761-s001.docx]

**SUPPLEMENTARY MATERIALS**

| **Diagnosis** | **Young** | **Adults** | **Middle-aged** | **Elderly** |
| --- | --- | --- | --- | --- |
| Alternative | -0.62 | 2.628 | -0.99 | -1.08 |
| Neuralgia | -1.07 | -1.67 | -1.55 | **4.33** |
| Other primary | 0.58 | -0.87 | -0.01 | 0.46 |
| Probable | 2.58 | 0.81 | -2.68 | 0.25 |
| Secondary | -2.15 | -2.35 | **4.19** | -0.75 |
| TACs | 0.02 | 1.72 | 0.31 | -2.15 |
| CTTH | 0.84 | -0.17 | -0.91 | 0.57 |
| ETTH | 0.16 | -0.54 | -0.88 | 1.44 |
| Non specified | 1.11 | -1.01 | -1.39 | 1.78 |

**Supplementary Table 1.** Adjusted standardized residuals from the post hoc analysis of non-migraine diagnoses across age groups. Residuals indicate the strength and direction of the association between each diagnosis and age group. Values greater than |3.20| are statistically significant after Bonferroni correction for multiple comparisons (*p* < 0.001389) and are shown in bold.

| **Diagnosis** | **Young** | **Adults** | **Middle-aged** | **Elderly** |
| --- | --- | --- | --- | --- |
| MWA | -0.51 | -1.04 | 1.85 | -0.59 |
| MA | **3.63** | **4.97** | **-4.03** | **-4.35** |
| CM | -2.33 | -2.79 | 1.11 | **4.17** |

**Supplementary Table 2.** Adjusted standardized residuals from the post-hoc analysis of migraine diagnoses across age groups. The table shows the analysis performed on 3,011 of the 3,013 patients with migraine; the two patients with migraine complications (both diagnosed with status migrainosus, ICHD-3 code 1.4.1) were excluded from this specific analysis. The contingency analysis included three mutually exclusive migraine diagnostic categories—migraine without aura (MWA), migraine with aura (MA), and chronic migraine (CM)—across the four age groups. The Bonferroni-adjusted significance level was calculated by dividing α = 0.05 by the 12 adjusted standardized residuals examined (α = 0.00417), corresponding to a two-sided critical value of |z| ≥ 2.87. Statistically significant residuals are shown in bold.

| Triggers (sexual acitivity/alcohol use/menstruation/relaxation/physical exertion/stress/sleep deprivation/ cough or defecation weather variations (Y) [N: 242] | 1/5/2/4/12/23/12/2/10 | 0/4/21/11/12/29/16/3(12 | 1/3/15/2/3/19/8/1/6 | 0/3/20/5/6/15/8/1/5 | 2/15/58/22/33/86/44/7/33 | Elderly: -3.21 for menstruation, 3.10 for physical exertion, 2.86 for stress; adult: -2.84 for physical exertion; young: 2.87 for menstruation |
| --- | --- | --- | --- | --- | --- | --- |
| Comorbidities (cardiological and cerebrovascular /neurological/psychiatric disorders/anxiety/endocrine and metabolic disease/hypothyroidism/ hypercholesterolemia/ neoplasms/ hypertension/previous smoking) [N: 235] | 2/3/12/10/20/1/12/6/14/6 | 3/2/15/5/22/9/4/10/17/2 | 1/2/8/5/7/0/2/1/5/0 | 1/1/5/2/8/2/0/0/1/1 | 7/8/40/22/57/12/18/17/37/9 | Elderly: 3.64 for anxiety, 4.03 for endocrine and metabolic disease, 5.73 for hypercholesterolemia, 3.56 for hypertension, 3.95 for previous smoking; middle-aged: 3.24 for hypothyroidism; adult: -3.19 for endocrine and metabolic disease; young: -3.01 for hypertension |

**Supplementary Table 3.** Categorical clinical variables in patients with migraine without aura (MWA), migraine with aura (MA), chronic migraine (CM) and migraine complications. Age groups are defined as follows: elderly (≥60 years), middle-aged (46–59 years), adults (31–45 years), and young (18–30 years). Data represent the number of subjects; percentages are reported in parentheses. The total number of subjects for each row is indicated in square brackets. Group differences were assessed using the chi-square test. Post hoc comparisons were performed using adjusted standardized residuals and corrected for multiple comparisons with the Bonferroni method.
